# Implications of vitamin D for flesh quality of grass carp (*Ctenopharyngodon idella*): antioxidant ability, nutritional value, sensory quality, and myofiber characteristics

**DOI:** 10.1186/s40104-023-00911-7

**Published:** 2023-09-27

**Authors:** Yao Zhang, Chaonan Li, Xiaoqiu Zhou, Weidan Jiang, Pei Wu, Yang Liu, Hongmei Ren, Lu Zhang, Haifeng Mi, Jiayong Tang, Ruinan Zhang, Lin Feng

**Affiliations:** 1 Animal Nutrition Institute, Sichuan Agricultural University, Chengdu, 611130 Sichuan China; 2 Fish Nutrition and Safety Production University Key Laboratory of Sichuan Province, Sichuan Agricultural University, Chengdu, 611130 China; 3 Key Laboratory for Animal Disease-Resistant Nutrition of China Ministry of Education, Sichuan Agricultural University, Chengdu, 611130 China; 4 Healthy Aquaculture Key Laboratory of Sichuan Province, Tongwei Co., Ltd., Chengdu, 610041 Sichuan China

**Keywords:** Antioxidant, Grass carp, Myofiber development, Nutritional value, Sensory quality, Vitamin D

## Abstract

**Background:**

Muscle represents a unique and complex system with many components and comprises the major edible part of animals. Vitamin D is a critical nutrient for animals and is known to enhance calcium absorption and immune response. In recent years, dietary vitamin D supplementation in livestock has received increased attention due to biological responses including improving shear force in mammalian meat. However, the vitamin D acquisition and myofiber development processes in fish differ from those in mammals, and the effect of vitamin D on fish flesh quality is poorly understood. Here, the influence of dietary vitamin D on fillet quality, antioxidant ability, and myofiber development was examined in grass carp (*Ctenopharyngodon idella*).

**Methods:**

A total of 540 healthy grass carp, with an initial average body weight of 257.24 ± 0.63 g, were allotted in 6 experimental groups with 3 replicates each, and respectively fed corresponding diets with 15.2, 364.3, 782.5, 1,167.9, 1,573.8, and 1,980.1 IU/kg vitamin D for 70 d.

**Results:**

Supplementation with 1,167.9 IU/kg vitamin D significantly improved nutritional value and sensory quality of fillets, enhancing crude protein, free amino acid, lipid, and collagen contents; maintaining an ideal pH; and reducing lactate content, shear force, and cooking loss relative to respective values in the control (15.2 IU/kg) group. Average myofiber diameter and the frequency of myofibers > 50 μm in diameter increased under supplementation with 782.5–1,167.9 IU/kg vitamin D. Levels of oxidative damage biomarkers decreased, and the expression of antioxidant enzymes and nuclear factor erythroid 2-related factor 2 signaling molecules was upregulated in the 1,167.9 IU/kg vitamin D treatment compared to respective values in the control group. Furthermore, vitamin D supplementation activated cell differentiation by enhancing the expression of myogenic regulatory factors and myocyte enhancer factors compared to that in the control group. In addition, supplementation with 1,167.9 IU/kg vitamin D improved protein deposition associated with protein synthesis molecule (target of rapamycin) signaling and vitamin D receptor paralogs, along with inhibition of protein degradation (forkhead box protein 1) signaling.

**Conclusions:**

Overall, the results demonstrated that vitamin D strengthened antioxidant ability and myofiber development, thereby enhancing nutritional value and sensory quality of fish flesh. These findings suggest that dietary vitamin D supplementation is conducive to the production of nutrient-rich, high quality aquaculture products.

**Supplementary Information:**

The online version contains supplementary material available at 10.1186/s40104-023-00911-7.

## Introduction

With the remarkable development of the aquaculture industry, global fisheries output has hit an all-time high in recent years [1]. Indeed, aquatic products have made significant contributions to global food security and nutrition in the 21^st^ century [2]. Currently, ensuring flesh quality is the greatest challenge facing the aquaculture industry, as poor quality hinders consumer acceptance and causes major economic losses for producers [3]. Flesh quality is affected by intrinsic and extrinsic factors, including fish species, rearing conditions, food sources, and nutrition. In terms of nutrition, dietary protein [4], fat [5], and minerals [6] have been shown to directly affect the nutritional value, tenderness, water-holding capacity, collagen synthesis, and antioxidant capacity of muscle tissue in grass carp, turbot, and blunt snout bream, respectively. These findings suggest that nutrients play an important role in regulating aquaculture products quality.

Vitamin D is an essential nutrient for animals [7]. As such, dietary vitamin D supplementation has been confirmed to improve oxidation and nutritional value in pork [8], enhance tenderness in beef [9], and maximize sensory quality in chicken [7]. Unlike mammals, which obtain vitamin D both through dietary consumption and ultraviolet solar radiation, fish maintain vitamin D levels primarily through diet, with levels accumulating throughout their lives [10]. This potentially allows for the nutritional strategy of vitamin D intervention to directly enhance fish flesh quality. Moreover, vitamin D mediates biological actions via the vitamin D receptor (VDR), which exhibits two unique paralogs, VDRa and VDRb, with individual functions in fish [10]. However, the correlation between vitamin D supplementation, VDR paralogs and fish flesh quality remains unclear thus far. Therefore, a detailed study was warranted to explore the specific effects of vitamin D on flesh quality.

Fish flesh quality is directly associated with antioxidant abilities and myofiber development [11]. Oxidative stress in fish muscle results in the damage to both the antioxidant system and flesh quality [12]. This damage is closely related to the excessive generation of reactive oxygen species (ROS), acceleration of oxidative damage, and decreased antioxidant enzyme expression [13]. There are numerous studies reporting positive consequences of vitamin D in terms of muscle antioxidant capacity, particularly in animals and human. In animal studies, dietary vitamin D_3_ supplementation decreased malondialdehyde (MDA) content in broiler chickens [7], and improved antioxidative capacity in the longissimus muscle of pigs [14]. Vitamin D_3_ in pig diets improved the total antioxidant activity and reduced lipid peroxidation in pork [15]. Pigs supplemented with vitamin D_3_ exhibited increased α-tocopherol content and decreased lipid peroxidation in the muscle [8]. Increased protein carbonyl (PC) content and decreased superoxide dismutase (SOD) and catalase (CAT) activities were observed in vitamin D-deficient rat muscle [16]. In human, vitamin D_3_ increased MnSOD and glutathione peroxidase (GPx) activities in the paraspinal muscles of patients with lower back pain [17]. Several recent studies have showed that vitamin D affects the antioxidant abilities in the intestines and hepatopancreas of aquatic animals [18, 19]. However, there are no studies examining the effects of vitamin D on muscle antioxidant capacity in fish. The content of unsaturated fatty acids in fish muscle is much higher than that of terrestrial animals, which is easy to cause oxidative damage, leading to the impaired muscle quality [20]. Therefore, the functions of vitamin D in muscle antioxidant ability in fish, as well as the underlying molecular mechanisms, remain largely unknown and require further study.

In most fish, muscle growth is indeterminate and myofiber development continues well beyond the juvenile stage; that is, flesh quality is continually modulated by deposition of nutrients, such as vitamin D [21]. Postembryonic myofiber development includes hyperplasia and hypertrophy, and the hypertrophy process has three main stages: proliferation of myoblasts, differentiation into myocytes that fuse to form multinucleated myotubes, and maturation of functional myofibers [21, 22]. Myocyte enhancer factors (MEFs) cooperate with myogenic regulatory factors (MRFs) to activate muscle-related gene expression, playing an important role in all stages of the differentiation process [23]. Meanwhile, myotube formation increases myofiber diameter and abundance, thus reflecting the importance of maintaining an optimal balance between protein synthesis and degradation [24]. In rats, vitamin D deficiency depressed growth and protein turnover in skeletal muscle [25], indicating an important role in myofiber development. However, the potential functions of vitamin D during myofiber development in fish have not been intensively studied.

As one of the most important economical species in China, grass carp (*Ctenopharyngodon idella*) is highly cultivated and consumed nationwide. The global production of grass carp in 2019 reached over 50 million tons [2]. Since living standards improve, consumer demands for high-quality aquatic products are steadily increasing. However, the flesh quality of cultured grass carp has declined due to high-density aquaculture in recent years [26]. To this end, the primary aim of the current study is to elucidate the role and mechanisms of vitamin D on flesh quality and myofiber development in grass carp. We hypothesize that vitamin D supplementation improves flesh quality and antioxidant abilities, while accelerating the myofiber development through hypertrophy and hyperplasia process in fish muscle. To test this hypothesis, we investigated the effects of vitamin D on nutritional quality, free amino acid profile, physicochemical properties, antioxidant enzymes, myofiber differentiation and protein deposition of fish muscle. To the best of our knowledge, our study is the first to investigate vitamin D and VDR paralogs as they pertain to fish flesh quality, myofiber development, and related molecular signaling. Our findings offer novel insights into the value of nutritional intervention with vitamin D and show that nutritionally optimized feeds have great potential to improve the quality of food products.

## Materials and methods

### Ethics statement

All experimental procedures were performed in accordance with the guidelines of the University of Sichuan Agricultural Animal Care Advisory Committee (China).

### Experimental diets

The composition of the basal diet is presented in Table S1 (Additional file 1). Protein sources primarily comprised gelatin, casein, and soybean protein concentrate, with linseed and soy oil as lipid sources. According to previous studies [27–33], graded levels of vitamin D_3_ (500,000 IU/g) were supplied at concentrations of 0 (un-supplemented control), 200, 400, 800, 1,200, and 2,000 IU/kg. The mineral and vitamin premixes were prepared via a stepwise expansion method. Dry ingredients were mixed and grounded through 80-mesh sieve. Once the vitamin D was fully blended with other ingredients, linseed oil and soy oil were further kneaded with the raw materials. All ingredients were added to distilled water and formed into pellets using an extruding pelletizer (EL-260, Youyi machinery factory, Shandong, China), and then air-dried in an air-conditioned room [34]. The diets were then stored in a freezer at −20 °C until use. Following the analysis procedures described by Zhang et al. [35], the final vitamin D levels in each treatment were determined to be 15.2 (un-supplemented control; VD15.2), 364.3 (VD364.3), 782.5 (VD782.5), 1,167.9 (VD1,167.9), 1,573.8 (VD1,573.8), and 1,980.1 (VD1,980.1) IU/kg feed.

### Experimental procedures

The experiment was carried out in our experimental base located in Dayi County of Chengdu. The fish selected for experiments were visibly healthy, with no parasites found under microscopic examination. Before the experiment, the fish were acclimatized for 4 weeks and fed 3 times daily at 7:00, 13:00, and 19:00. After adaptation, the fish were provided with an un-supplemented basal diet for 2 weeks to reduce the pre-accumulated levels of vitamin D. Subsequently, a total of 540 fish (initial weight: 257.24 ± 0.63 g) were randomly allocated into 18 underwater cages (1.4 m × 1.4 m× 1.4 m; 30 fish each) representing 6 experimental treatments (3 cages/treatment; 90 fish/treatment). All aquaculture net cages were located in freshwater ponds with microporous aeration designed specifically for aquaculture. The physicochemical properties of the water were monitored regularly to ensure consistent rearing conditions. The dissolved oxygen concentration, water temperature, and pH were maintained at > 6 mg/L, 28.0 ± 2.1 °C and 7.0 ± 0.2, respectively. Fish were subject to natural light conditions and fed to satiation 3 times daily for 70 d.

### Sampling

After the feeding trial, 15 randomly selected fish from each treatment (5 fish each replicate) were euthanized in a benzocaine bath (50.0 mg/L). Fish muscle (above the lateral line and on the left side behind the head) was removed immediately. Six samples per treatment (2 fish each replicate) were preserved at −80 °C for biochemical assay, real-time quantitative polymerase chain reaction (qPCR), and Western blot analyses. Tissue samples from an additional 6 fish in each treatment (2 fish each replicate) were preserved in 4% paraformaldehyde for hematoxylin and eosin (H&E) staining and immunofluorescence analyses, and those of another 3 fish from each treatment (1 fish each replicate) were fixed with glutaraldehyde buffer for transmission electron microscopy.

### Body composition and biochemical analysis

The approximate fish fillet compositions were analyzed according to previously published protocols [34]. Moisture, crude protein, and ash content were analyzed via vacuum freeze drying (GZX-9240MBE, Shanghai, China), the Kjeldahl method with sulfuric acid digestion, and heating to constant mass in a muffle furnace (Taisete Co., Ltd., Tianjin, China), respectively. The crude lipid levels were assessed according to the Soxhlet extraction method [20]. The shear force and cooking loss of flesh were analyzed immediately after sample collection following previously reported procedures [11]. Muscle pH value; lactate, hydroxyproline, and collagen content; cathepsin B/L activity; and amino acid profiles were determined using previously published protocols [4].

Antioxidant enzyme activities were assessed was as follows. Muscle tissues were ground with liquid nitrogen, diluted with physiological saline solution, and centrifuged at 6,000 × *g* for 20 min at 4 ºC to collect the supernatant. Nanjing Jiancheng commercial assay kits (Nanjing, China) were used to evaluate the activities of ROS, PC, MDA, total antioxidant capacity (T-AOC), SOD, CAT, GPx, glutathione reductase (GR), glutathione S-transferases (GST), glutathione (GSH), anti-superoxide anion (ASA), and anti-hydroxyl radical (AHR) according to manufacturer’s instructions. The information regarding commercial kits is presented in Table S2.

### Transmission electron microscopy

Muscle samples (0.1 cm × 0.1 cm × 0.1 cm) were fixed in glutaraldehyde and rinsed with 0.1 mol/L phosphate buffer thrice for 15 min. Samples were then fixed in 1% osmium tetroxide for 2 h, dehydrated serially in 30%–100% ethyl alcohol and dehydrated further with 100% acetone twice. The samples were then treated with acetone and Epon 812 (1:1; SPI Supplies, West Chester, PA, USA) for 4 h, incubated with acetone and Epon 812 (1:2) overnight, and embedded in pure Epon 812 for 5 h. Samples were then embedded in Epon 812 at 37 °C overnight, polymerized at 60 °C for 48 h, and sliced into 80 nm sections with an ultra-microtome (EM UC7, Leica, Wetzlar, Germany) before placing on cuprum grids. Cuprum grids were stained with uranium acetate and lead citrate and dried overnight at approximately 20 °C. Images were obtained using a JEM-2100Plus transmission electron microscope (JEOL, Tokyo, Japan) at 80 kV acceleration voltage and were recorded on Kodak Electron Image Film SO-163 (Eastman Kodak Co., Rochester, NY, USA).

### Tissue embedding, H&E staining, and immunofluorescence

Muscle sections (0.5 cm × 0.5 cm × 1 cm) were embedded in molds with Tissue-Tek® O.C.T. Compound (Sakaru, Tokyo, Japan), cooled with liquid nitrogen, and sliced into 4 μm sections with a freezing microtome (CM1950, Leica, Wetzlar, Germany). Prepared samples were placed on positively charged microscope slides for H&E and immunofluorescence analyses.

Specimens were stained with H&E and then visualized under an Olympus BX43 light microscope (Olympus, Tokyo, Japan). Three fields of view (0.35 mm × 0.35 mm) were randomly selected from each sample to assess myofiber morphological characteristics.

For immunofluorescence staining, antigens were repaired with antigen retrieval solution (Beyotime, Nanjing, China), treated with 3% hydrogen peroxide, blocked in blocking buffer (5% goat serum, 2% bovine serum albumin, and 0.2% Triton X-100), and incubated with primary antibodies overnight at 4 °C. Slides were rewarmed to approximately 20 °C under ambient conditions and incubated with immunofluorescent secondary antibodies for 2 h. Subsequently, the slides were stained with DAPI and covered with cover slides. Immunofluorescence images were obtained using a DMI4000B inverted fluorescence microscope (Leica). Antibody information is listed in Table S3.

### qPCR

Muscle RNA extraction, reverse transcription, and qPCR were performed following the procedures established for our laboratory [35]. RNA was extracted using RNAiso Plus (Takara Bio, Kusatsu, Japan), treated with DNase I (Beyotime), and analyzed for purity and quantity via agarose gel electrophoresis and spectrophotometry (A_260/280_ ratio), respectively. Reverse transcription was performed using the PrimeScript™ RT Reagent Kit (Takara Bio). The reaction system for qPCR contained 1 µL complementary DNA, 0.2 µL ROX, 5 µL SYBR Premix Ex Taq, 0.4 µL primers, and 3.4 µL nuclease-free water (Vazyme, Nanjing, China). The reaction was run in a QuanStudio 5Real-Time System (Life Technologies, Carlsbad, CA, USA) according to manufacturer protocol. The oligonucleotide primers used are presented in Table S4. The 2^−ΔΔCT^ method was used to calculate the changes in target gene expression relative to β-actin levels.

### Western blotting and gel electrophoresis

The homogenized muscle tissue was extracted in lysis solution (RIPA:PMSF = 89:1) for 30 min and centrifuged at 1,000 × *g* for 15 min at 4 °C. The liquid supernatant was extracted and treated with 5× loading buffer (Beyotime). The protein concentration of the samples was determined using a BCA kit (Beyotime) with denaturing at 96 °C for 8 min. Protein samples from fish muscle with equal mass (40 µg/lane) were extracted, separated on sodium dodecyl sulfate-glycine polyacrylamide gel, and then transferred to a polyvinylidene fluoride membrane. These membranes were blocked with bovine serum albumin (5%) for 2 h and incubated with primary antibodies overnight at 4 °C. Subsequently, membranes were incubated with secondary antibodies and immune complexes were visualized using a ChemiDoc imaging system (Bio-Rad, Hercules, CA, USA). Information on primary and secondary antibodies, including dilution ratios, is presented in Table S5.

### Statistical analyses

Before statistical analysis, all experimental data in the present study were tested for homogeneity and normal distribution using Levene’s test and the Shapiro-Wilk test, respectively. Data were analyzed using one-way analysis of variance and Duncan’s multiple range test to evaluate statistical differences among treatments at the significance level of *P* < 0.05 in SPSS Statistics v.25 (IBM Corp., Armonk, NY, USA). Unpaired Student’s *t*-tests were used to analyze sarcomere lengths at the significance level of *P* < 0.05. Image J 1.8.0 (NIH, Bethesda, MD, USA) was applied to measure myofiber diameter and sarcomere length. The appropriate dietary vitamin D supplementation level was estimated using a quadratic regression model according to Zhang et al. [35]. GraphPad Prism 8.0 software (GraphPad Software, Inc.) was used for data visualization.

## Results

### Nutrient composition and physicochemical properties of fish fillets

**Fig. 1:**
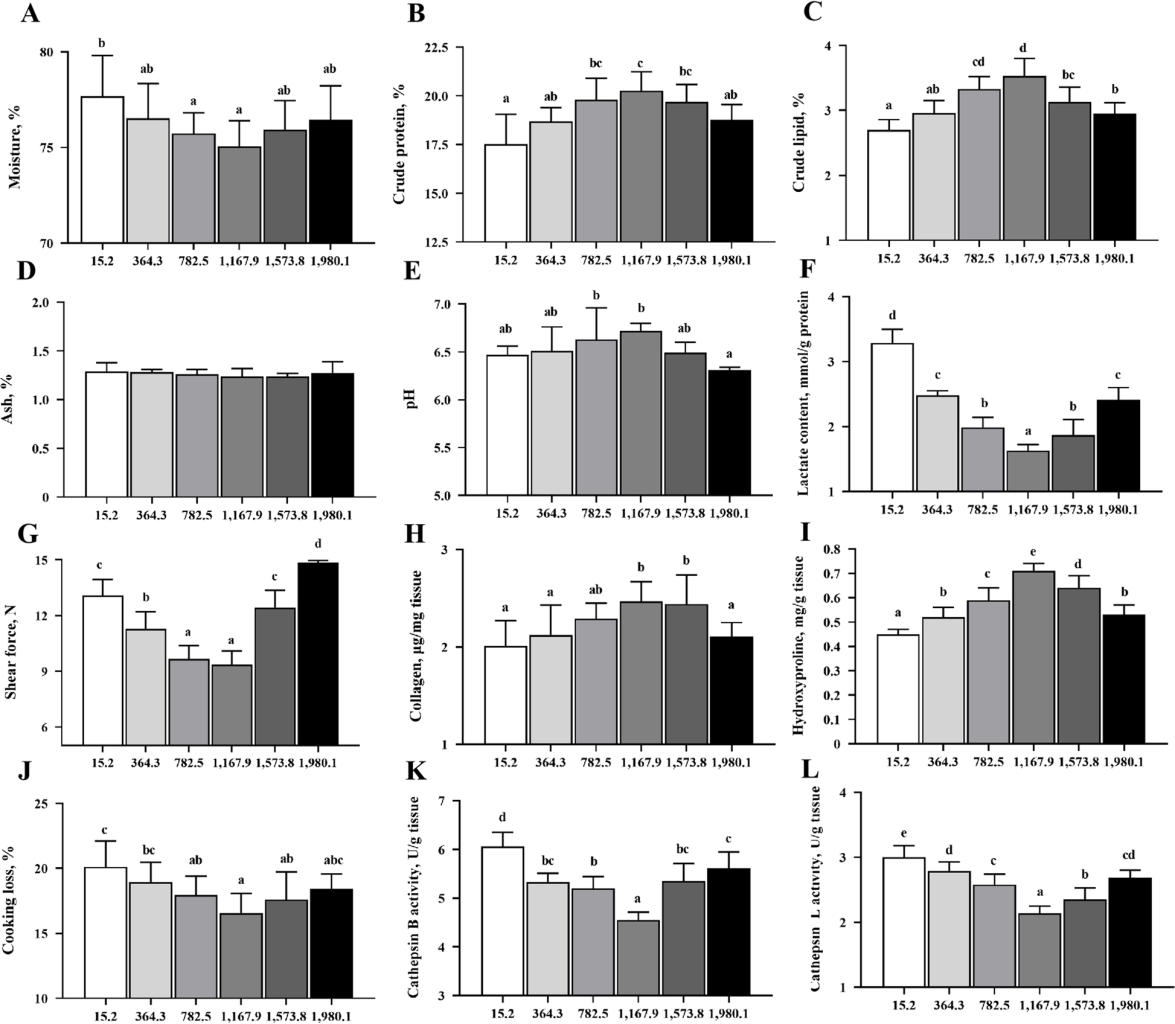
Proximate composition and physicochemical properties of the muscle from grass carp (*Ctenopharyngodon idella*) after a 70-day growth trial. **A** Moisture content; **B** Crude protein content; **C** Crude lipid content; **D** Ash content; **E** pH; **F** Lactate content; **G** Shear force; **H** Collagen content; **I** Hydroxyproline level; **J** Cooking loss; **K** Cathepsin B activity; **L** Cathepsin L activity in fillets. Results are shown as means ± SD (*n* = 6). Different letters above bars indicate significant differences (*P* < 0.05)

The nutrient composition and physicochemical properties of fillets under varying vitamin D supplementation levels were assessed (Fig. 1). Compared those under the un-supplemented control treatment, fillets from the VD1,167.9 treatment group exhibited significantly increased crude protein and lipid contents, as well as decreased moisture content (*P* < 0.05). No significant difference was observed in ash content between any treatments (*P* > 0.05). In the sensory quality analysis, optimal vitamin D supplementation (1,167.9 IU/kg) yielded significantly lower lactate levels, shear strength, and cooking loss than the control values, along with significantly higher pH, collagen, hydroxyproline levels (*P* < 0.05). Cathepsin B/L levels were lowest in the VD1,167.9 group. These findings suggested that vitamin D supplementation enhanced the nutritional value and sensory quality of fish flesh.

Muscle amino acid profiles under dietary vitamin D treatments are displayed in Table 1. Compared with corresponding values in the un-supplemented control group, the contents of aspartic acid, glutamic acid, glycine, and arginine enhanced significantly (*P* < 0.05) with vitamin D administration up to 1,167.9 IU/kg, and then decreased at higher vitamin D levels. Alanine, serine, valine, isoleucine, leucine, tyrosine contents all reached maximum values at a vitamin D concentration of 782.5 IU/kg. The VD1,573.8 group had the highest levels in threonine, lysine, and histidine among all treatments. Methionine, phenylalanine, and proline contents significantly increased with vitamin D supplementation (*P* < 0.05), and the highest levels were observed in the VD1,980.1 treatment.

**Table 1:** Effects of dietary vitamin D on muscle amino acids profile in on-growing grass carp^1^

Item^1^, mg/100g tissue	Vitamin D level, IU/kg diet					
	15.2 (control)	364.3	782.5	1,167.90	1,573.80	1,980.10
Aspartic acid	1.85 ± 0.12^a^	2.33 ± 0.22^bc^	2.41 ± 0.20^bc^	2.56 ± 0.22^c^	2.23 ± 0.19^b^	1.92 ± 0.20^a^
Glutamic acid	4.41 ± 0.32^a^	5.26 ± 0.51^b^	6.69 ± 0.36^d^	7.48 ± 0.57^e^	5.91 ± 0.50^c^	5.86 ± 0.44^c^
Alanine	11.15 ± 0.84^a^	12.98 ± 0.68^bc^	14.72 ± 1.03^d^	13.68 ± 1.21^ cd^	12.53 ± 1.19^bc^	11.85 ± 0.65^ab^
Serine	3.70 ± 0.30^a^	4.33 ± 0.39^b^	4.98 ± 0.28^c^	4.14 ± 0.24^b^	4.04 ± 0.17^b^	3.49 ± 0.20^a^
Glycine	16.28 ± 0.93^a^	18.30 ± 1.08^b^	20.92 ± 1.54^c^	22.55 ± 1.15^d^	19.38 ± 1.69^bc^	18.48 ± 1.63^b^
Threonine	10.50 ± 0.67^a^	11.78 ± 0.89^b^	12.91 ± 0.81^ cd^	13.64 ± 0.64^d^	14.74 ± 0.32^e^	12.27 ± 0.72^bc^
Valine	3.10 ± 0.26^a^	3.85 ± 0.13^b^	4.27 ± 0.31^d^	3.81 ± 0.27^e^	3.45 ± 0.33^d^	3.30 ± 0.29^c^
Methionine	2.44 ± 0.13^a^	3.18 ± 0.18^b^	4.95 ± 0.38^c^	5.56 ± 0.37^d^	6.08 ± 0.34^e^	6.52 ± 0.39^f^
Isoleucine	2.94 ± 0.24^a^	3.55 ± 0.21^bc^	4.35 ± 0.25^d^	3.82 ± 0.15^c^	3.57 ± 0.32^bc^	3.32 ± 0.27^b^
Leucine	3.57 ± 0.25^a^	4.45 ± 0.32^bc^	5.12 ± 0.41^d^	4.63 ± 0.27^c^	4.22 ± 0.31^b^	3.73 ± 0.24^a^
Phenylalanine	3.13 ± 0.25^a^	3.82 ± 0.30^b^	3.98 ± 0.20^b^	4.10 ± 0.20^b^	4.62 ± 0.23^c^	4.79 ± 0.42^c^
Lysine	36.13 ± 2.43^a^	38.14 ± 3.59^ab^	40.37 ± 2.84^b^	44.90 ± 3.39^c^	48.87 ± 4.00^d^	45.04 ± 2.35^c^
Histidine	93.14 ± 6.11^a^	97.08 ± 9.40^a^	102.11 ± 8.39^ab^	110.58 ± 7.30^b^	134.89 ± 7.50^c^	127.39 ± 8.48^c^
Arginine	17.40 ± 1.38^a^	19.64 ± 0.87^b^	21.27 ± 1.72^b^	23.78 ± 2.24^c^	21.35 ± 0.78^b^	19.69 ± 1.51^b^
Proline	39.84 ± 2.38^a^	43.27 ± 3.45^b^	47.33 ± 4.08^c^	47.11 ± 2.75^c^	47.30 ± 1.21^c^	47.43 ± 3.68^c^
Tyrosine	3.77 ± 0.29^a^	4.27 ± 0.32^bc^	4.83 ± 0.44^d^	4.34 ± 0.36^c^	4.11 ± 0.30^abc^	3.86 ± 0.27^ab^

^1^Values are shown as means ± SD

^a–e^Different superscripts indicate significant differences among different vitamin D levels (*P* < 0.05)

### Antioxidant parameters of fish muscle

**Fig. 2:**
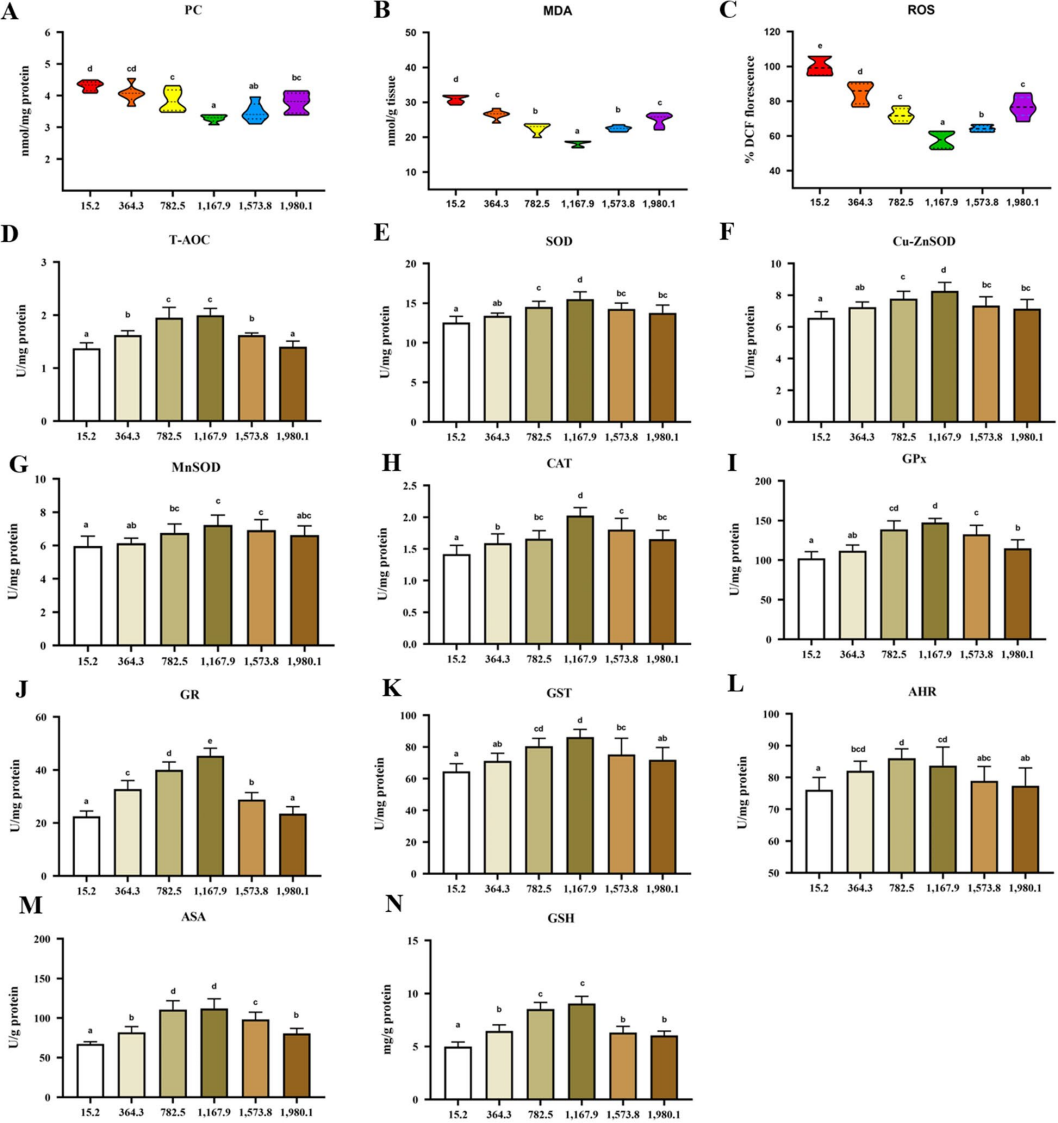
Effects of vitamin D on the activities of antioxidant enzymes and glutathione (GSH) in the muscle of grass carp (*n* = 6). **A** Protein carbonyl (PC); **B** Malondialdehyde (MDA); **C** Reactive oxygen species (ROS); **D** Total antioxidant capacity (T-AOC); **E** Superoxide dismutase (SOD); **F** CuZnSOD; **G** MnSOD; **H** Catalase (CAT); **I** Glutathione peroxidase (GPx); **J** Glutathione reductase (GR); **K** Glutathione S-transferase (GST); **L** Antihydroxyl radical (AHR); **M** Anti-superoxide anion (ASA); **N** GSH. Values are shown as means ± SD. Results with different superscripts are significantly different (*P* < 0.05)

The oxidative damage biomarker indices and key antioxidative enzyme activities were analyzed to determine the effect of vitamin D on muscle antioxidant capacity (Fig. 2A–N). The control group displayed the highest levels of oxidative damage biomarkers (MDA, PC and ROS), and these values significantly decreased with the increase in vitamin D supplementation (*P* < 0.05; Fig. 2A–C). The PC, MDA and ROS reached a minimum at VD1,167.9 group.

As shown in Fig. 2D–N, the activities of T-AOC, SOD, CuZnSOD, MnSOD, CAT, GPx, GR, GST, ASA, and GSH significantly increased with vitamin D supplementation and reached the highest values in the VD1,167.9 group (*P* < 0.05). The value of AHR was the highest in the VD782.5 group, and subsequently decreased with a further increase in vitamin D supplementation.

**Fig. 3:**
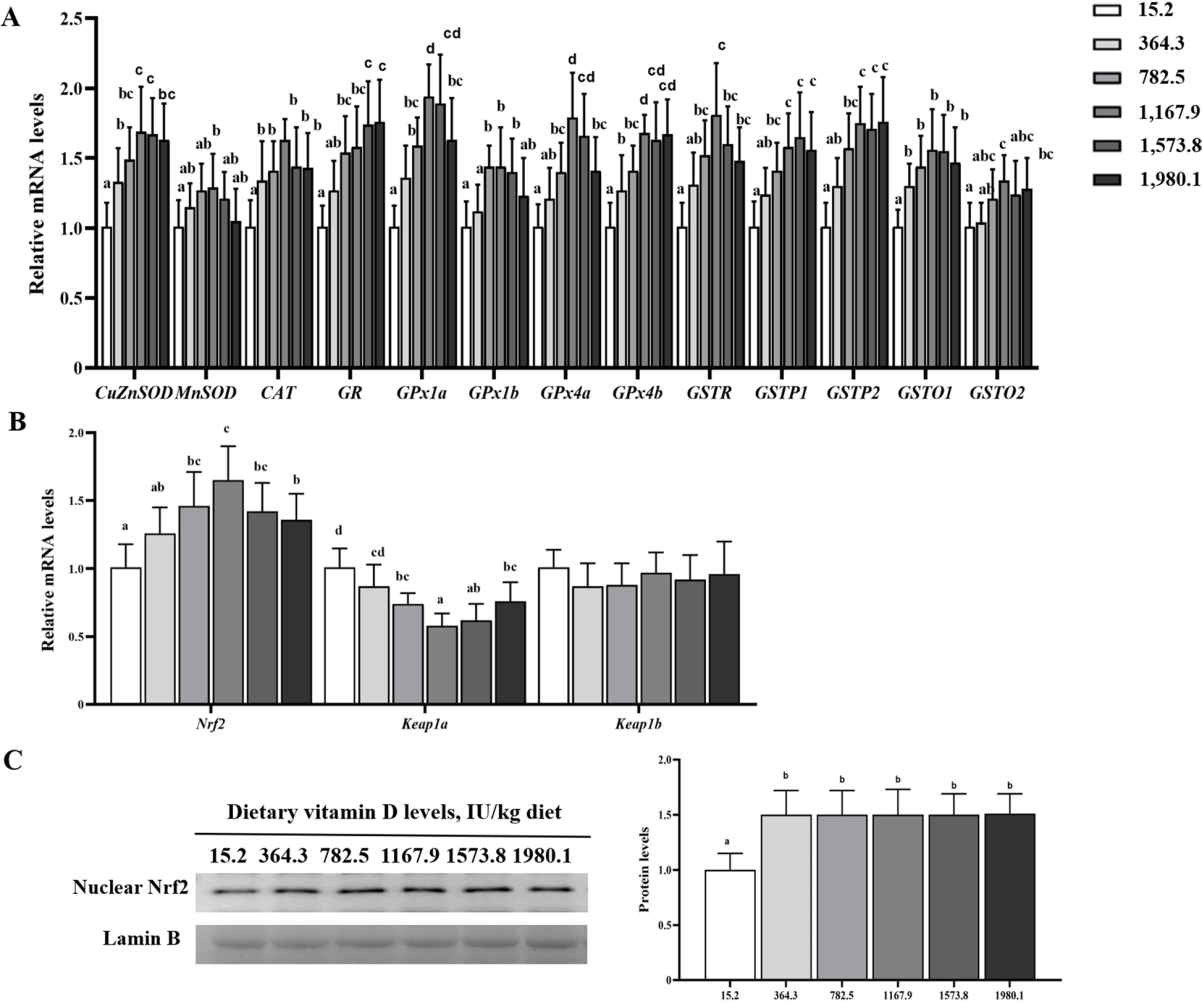
Effects of vitamin D on gene and protein levels of antioxidant parameters in the muscle of grass carp (*n* = 6). **A** Relative mRNA levels of antioxidant enzymes; **B** Relative mRNA levels of NF-E2-related factor 2 (Nrf2) signaling; **C** Protein level of Nrf2. Values are shown as means ± SD. Results with different superscripts are significantly different (*P* < 0.05)

The effects of dietary vitamin D on muscle antioxidant-related parameters are displayed in Fig. 3. Dietary vitamin D supplementation upregulated transcript levels of antioxidant enzymes (*P* < 0.05; Fig. 3A). The mRNA and protein levels of key antioxidant regulator molecule (nuclear factor erythroid 2-related factor 2; Nrf2) were enhanced in vitamin D supplementation groups (*P* < 0.05; Fig. 3B and C). Kelch-like ECH-associated protein 1a (Keap1a) expression was down-regulated with vitamin D supplementation, and the minimum value was seen in the VD1,167.9 group. Vitamin D supplementation did not affect the mRNA levels of Kea1b (*P* > 0.05). Overall, vitamin D supplementation strengthened antioxidant abilities in fish muscle.

### Morphological characteristics of fish fillet myofibers

**Fig. 4:**
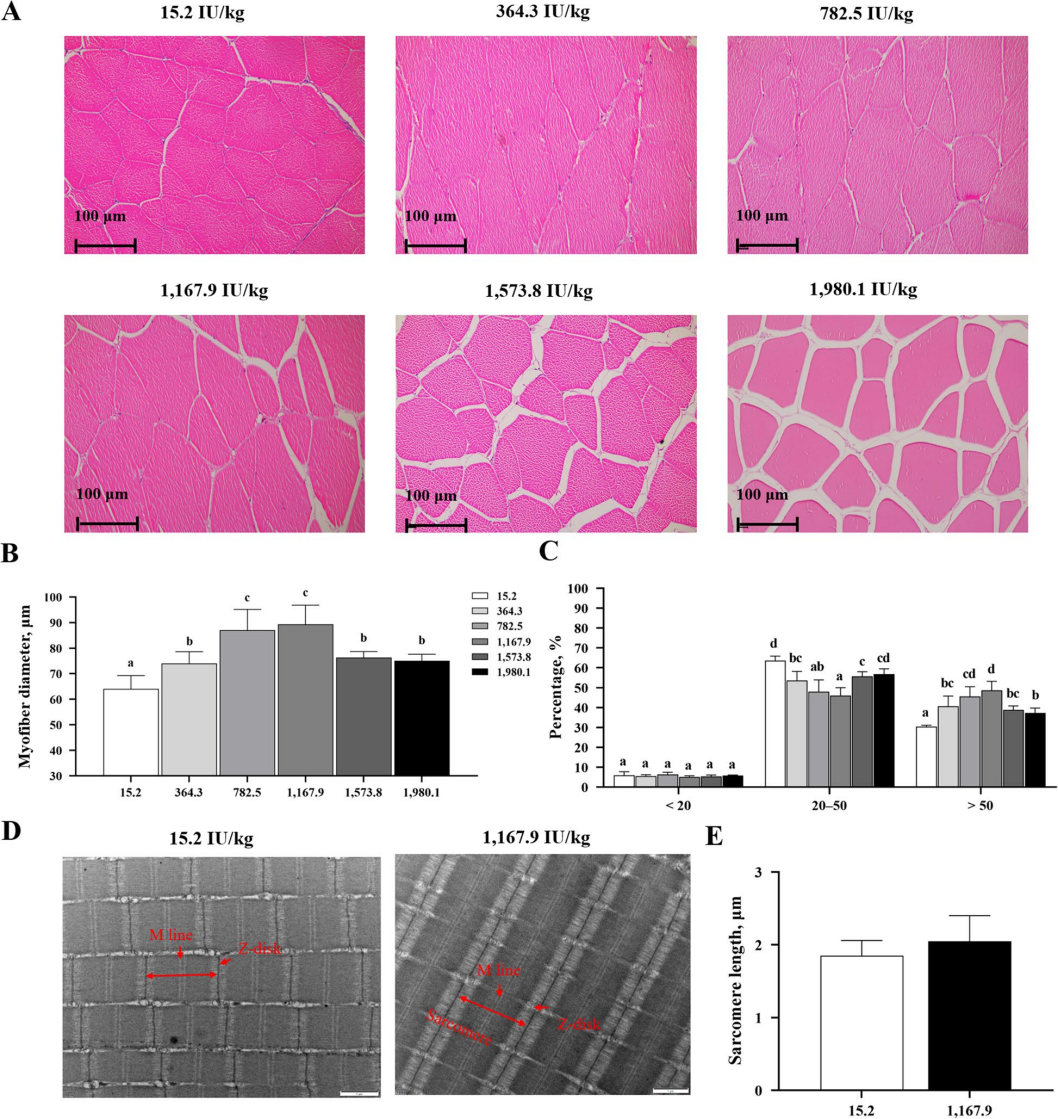
Histological observation of myofibers in grass carp fed different levels of vitamin D. **A** Hematoxylin-eosin staining results in cross-sections of fish muscle (200×; *n* = 6); **B** Myofiber diameter; **C** Frequency of distribution (%) of myofibers in different diameter classes; **D** Transmission electron microscope image; **E** Sarcomere length of myofibers in fish supplemented with 15.2 (control) and 1,167.9 IU/kg vitamin D (*n* = 3). M line, Z disk, and sarcomere are shown to represent myofiber structure. Values are shown as means ± SD. Different letters above bars indicate significant differences (*P* < 0.05)

Histological analysis revealed that vitamin D treatment increased average myofiber diameter compared to that of the un-supplemented control (Fig. 4A and B). The VD1,167.9 group exhibited much larger myofibers than fish in the other treatment groups (*P* < 0.05). Meanwhile, the VD15.2 group exhibited significantly more myofibers measuring 20–50 μm in diameter but fewer measuring over 50 μm than in the vitamin D supplemented groups. The abundance of small-diameter myofibers (< 20 μm) was consistent across all experimental groups (*P* > 0.05; Fig. 4C). Sarcomere length was also unaffected by vitamin D treatment (*P* > 0.05; Fig. 4D and F).

### MRFs and MEFs in fish muscle

**Fig. 5:**
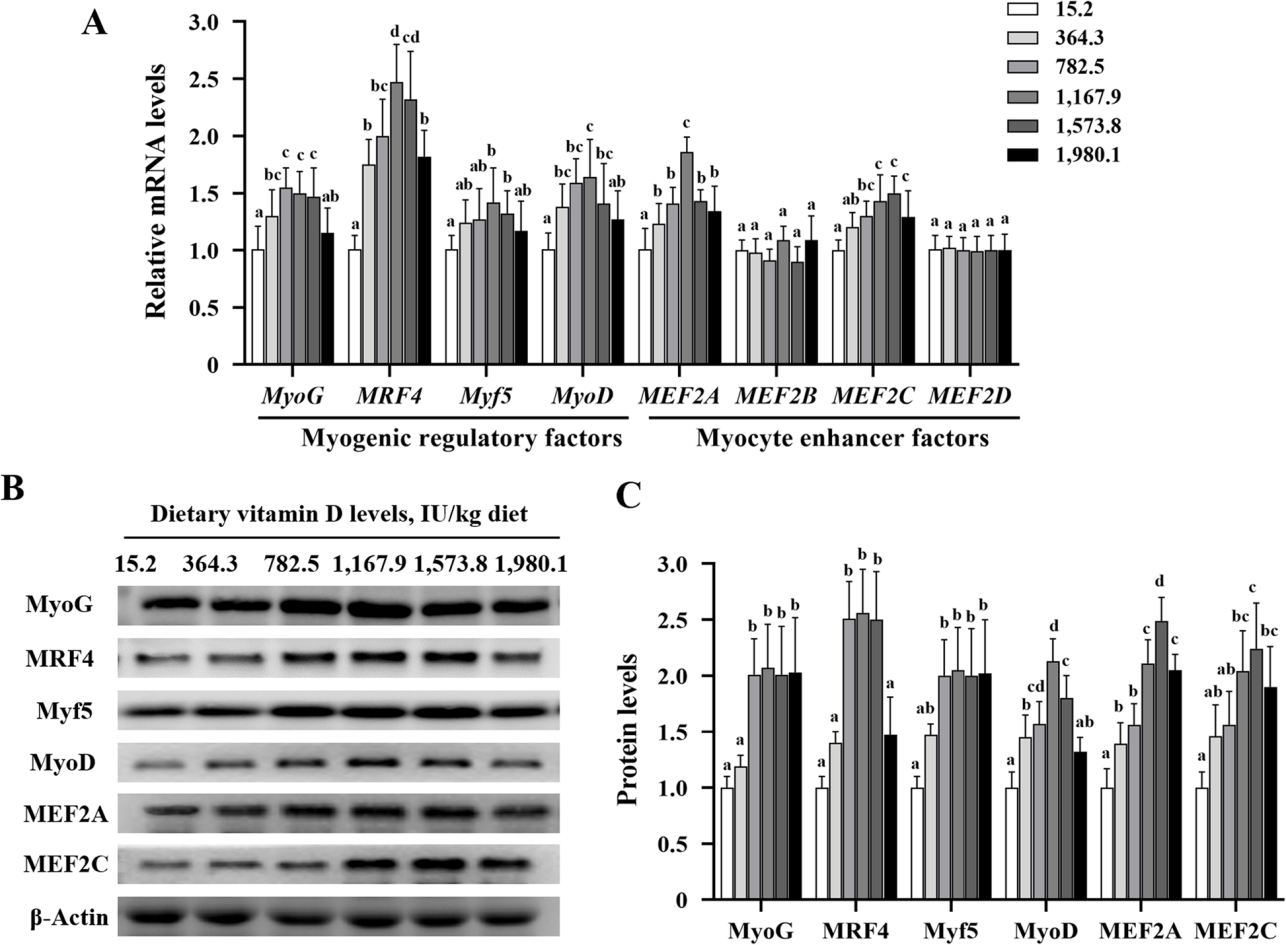
Effects of vitamin D on gene (**A**) and protein levels (**B** and **C**) of myogenic regulatory factors and myocyte enhancement factors in the muscle of grass carp (*n* = 6). Values are shown as means ± SD. Results with different superscripts are significantly different (*P* < 0.05). MyoD, myogenic differentiation antigen; MRF4, myogenic regulatory factor 4; Myf5, myogenic factor 5; MyoG, myogenin; MEF, myocyte enhancer factor

Vitamin D supplementation in grass carp upregulated transcript levels of myogenin (*MyoG*), myogenic regulatory factor 4 (*MRF4*), myogenic factor 5 (*Myf5*), myogenic differentiation antigen (*MyoD*), *MEF2A*, and *MEF2C* relative to control values (*P* < 0.05; Fig. 5A). Optimal vitamin D supplementation (1,167.9IU/kg) also increased the protein levels of MyoG, MRF4, Myf5, and MyoD (*P* < 0.05) in comparison with control levels. MEF2A and MEF2C reached the highest values in the VD1,573.8 group. However, vitamin D supplementation did not affect the mRNA or protein levels of MEF2B or MEF2D (*P* > 0.05; Fig. 5B and C). Overall, vitamin D supplementation appeared to promote myofiber development by facilitating the expression of MRFs and MEFs.

### Protein synthesis–related signaling in fish muscle

**Fig. 6:**
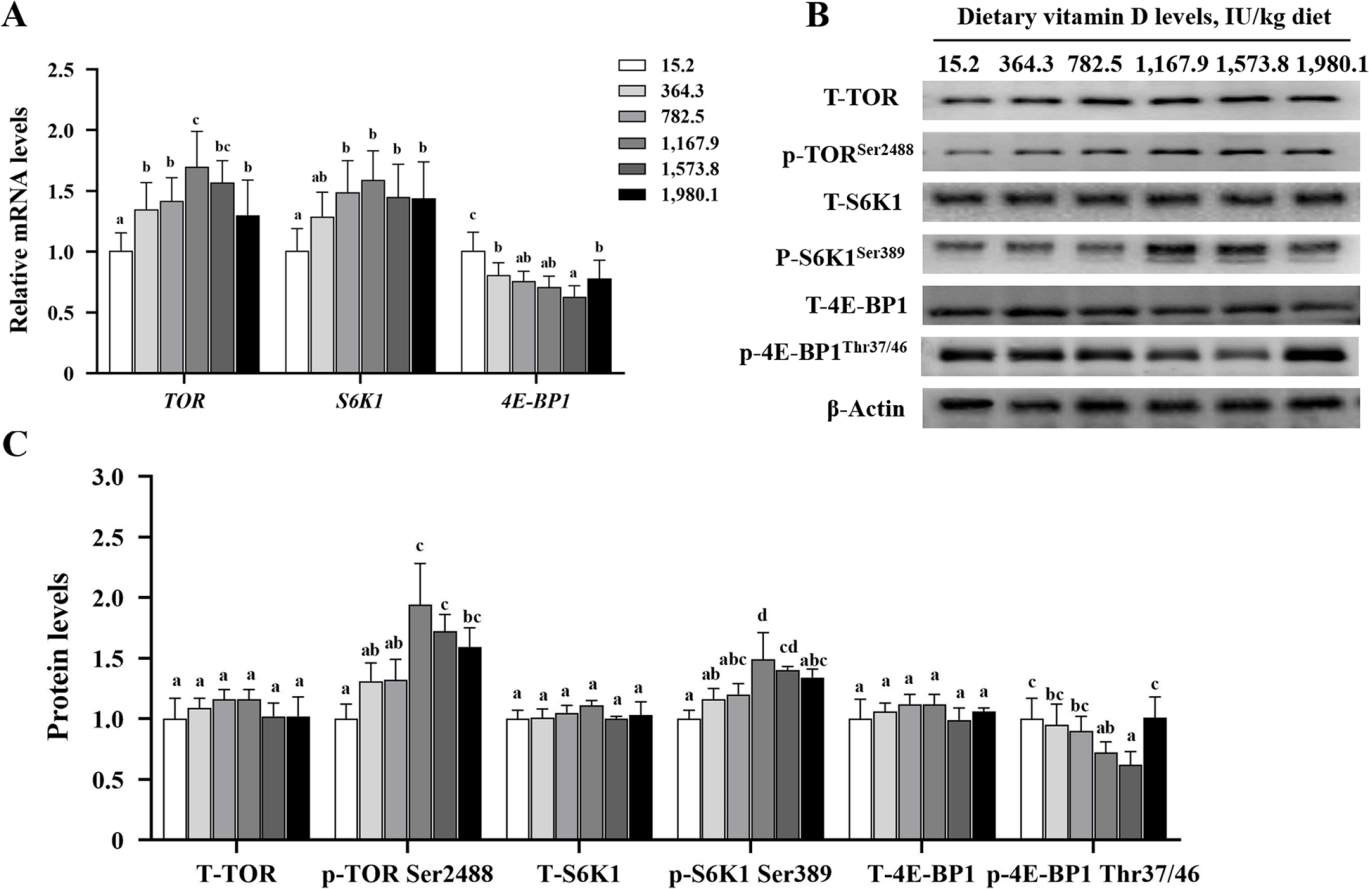
Effects of vitamin D on the transcription (**A**) and protein levels (**B** and **C**) of protein synthesis signaling molecules in the muscle of grass carp (*n* = 6). Values are shown as means ± SD. Results with different superscripts are significantly different (*P* < 0.05). TOR, target of rapamycin; S6K1, ribosomal protein S6 kinase; 4E-BP1, eIF4E-binding protein 1

Dietary vitamin D administration markedly elevated mRNA expression of target of rapamycin (*TOR*) and ribosomal protein S6 kinase 1 (*S6K1*) and significantly reduced transcription levels of 4E-binding protein 1 (*4E-BP1*; *P* < 0.05; Fig. 6A) when compared with un-supplemented control values. The protein levels of p-TORSer488 and p-S6K1Ser389 were enhanced with vitamin D supplementation up to VD1,167.9 group but decreased with higher vitamin D levels. Protein levels of p-4E-BP1Thr37/46 exhibited the lowest values in the VD1,573.8 groups, whereas those of T-TOR, T-S6K1, and T-4E-BP1 were not affected by dietary vitamin D (*P* > 0.05; Fig. 6B and C). These data indicated that vitamin D increased protein synthesis in fish muscle.

### Protein degradation–related signaling in fish muscle

**Fig. 7:**
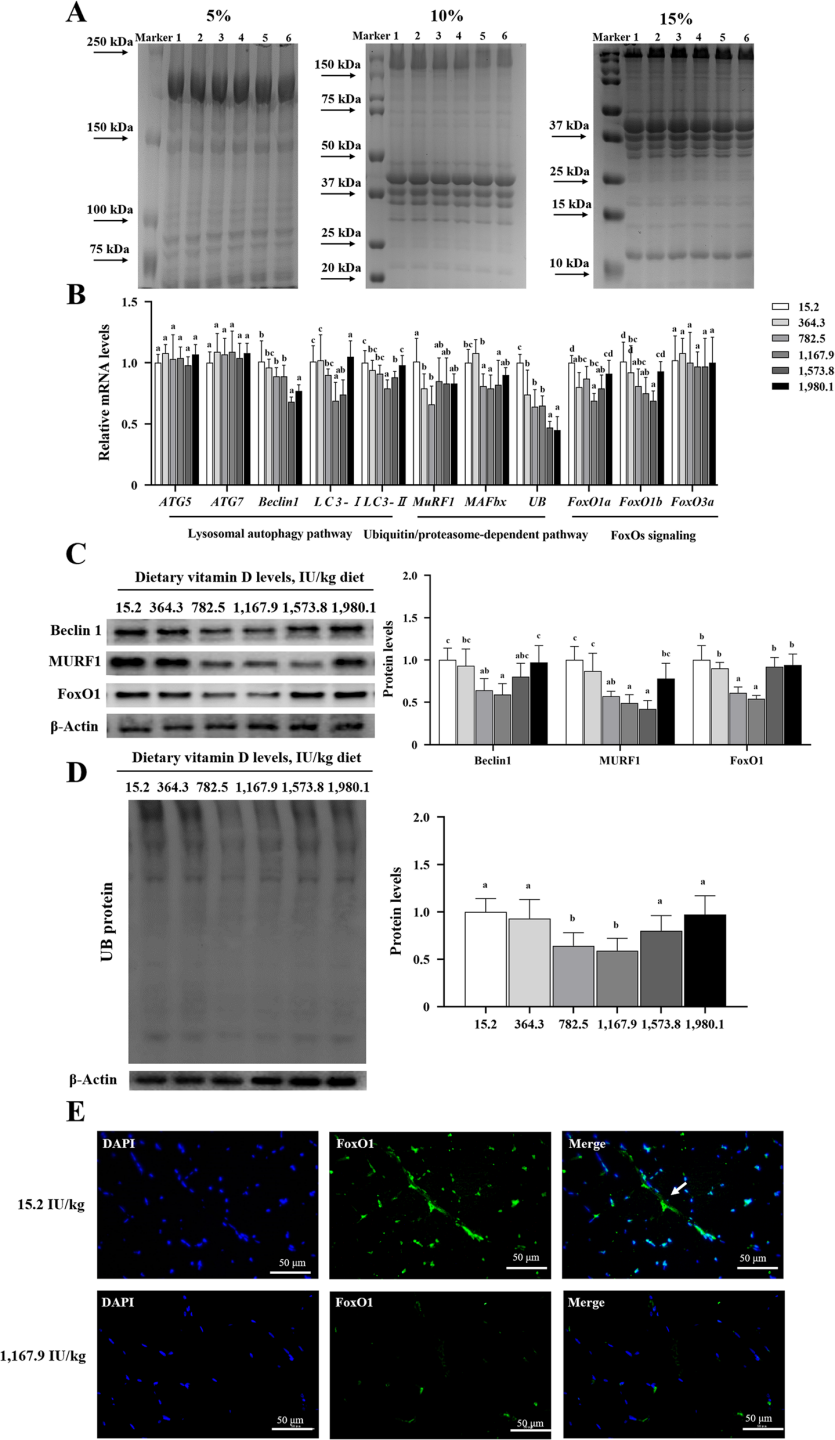
Effects of vitamin D on protein degradation in the muscle of grass carp. **A** SDS-PAGE results of muscle protein extract in different concentration separation gel. M, marker; numbers 1–6 represent different vitamin D levels from 15.2 to 1,980.1 IU/kg (*n* = 3). **B** and **C** Effects of dietary vitamin D on the transcription (**B**) and protein levels (**C**) of autophagy-lysosome pathway, ubiquitin-proteasome pathway, and FOXO molecules (*n* = 6). **D** UB protein level. **E** Immunofluorescence results of FoxOX1 in the 15.2 and 1,167.9 IU/kg groups (*n* = 3). The white arrow shows nuclear co-localization. Values are shown as means ± SD. Results with different superscripts are significantly different (*P* < 0.05). ATG, autophagy-related gene; LC3, light chain 3; MuRF1, muscle-specific ring finger; MAFbx, muscle atrophy F-box; UB, ubiquitin; FoxO, forkhead box O

SDS-PAGE showed that protein degradation–related factors remained consistent across treatments (Fig. 7A). Relative mRNA expression of *Beclin1*, light chain (*LC*)*3-I*, *LC3-II*, muscle-specific ring finger 1 (*MuRF1*), muscle atrophy F-box (*MAFbx*), forkhead box O (*FoxO*)*1a*, and *FoxO1b* first decreased with increasing dietary vitamin D levels (*P* < 0.05), and then exhibited an increasing trend (Fig. 7B). *Beclin1* and *FoxO1b* mRNA levels in the VD1,573.8 groups were the lowest of all treatments (*P* < 0.05). A significant reduction in *LC3-I*, *LC3-II*, *MAFbx*, and *FoxO1a* was observed in the VD1,167.9 groups relative to values in the control group, with levels tending to increase under higher vitamin D levels. The minimum *MuRF1* mRNA level was observed in the 782.5 IU/kg group. Ubiquitin (*UB*) mRNA levels were significantly lower in all vitamin D supplemented groups than in the control. However, the expression of autophagy related 5 (*ATG5*), *ATG7*, and *FoxO3a* remained consistent across treatments (*P* > 0.05). Vitamin D supplementation decreased the protein levels of Beclin1 and FoxO1, and the lowest values was observed in the VD1.167.39 group. MuRF1 protein levels were lowest in the VD1,573.8 group (Fig. 7C), whereas the UB protein level was much lower in the 782.5 and 1,167.9 IU/kg groups than in the control (*P* < 0.05; Fig. 7D). Immunofluorescence results confirmed reduced nuclear translocation of FoxO1 in the VD1,167.9 group (Fig. 7E). These data indicated that vitamin D inhibited protein degradation factors in fish muscle.

### VDR signaling in fish muscle

**Fig. 8:**
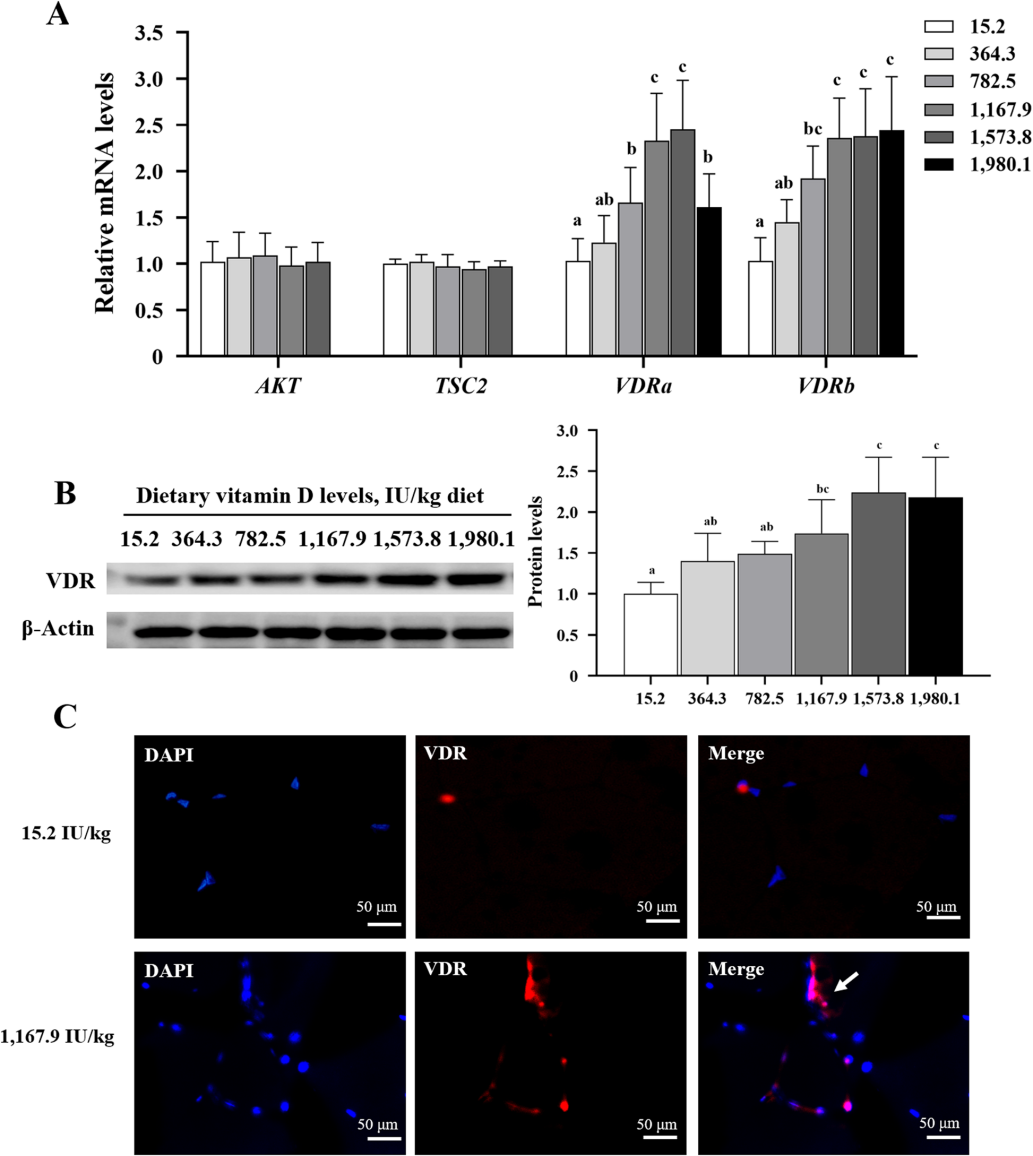
Effects of vitamin D on the transcription levels of AKT, TSC2, and VDR (**A**), and on protein levels of VDR (**B**) in the muscles of grass carp (*n* = 6). **C** Immunofluorescence results for VDR in 15.2 and 1,167.9 IU/kg treatment groups. The white arrow shows nuclear co-localization (*n* = 3). Values are shown as means ± SD. Results with different superscripts are significantly different (*P* < 0.05). AKT, protein kinase B; TSC2, tuberous sclerosis 2; VDR, vitamin D receptor

As shown in Fig. 8A, vitamin D supplementation did not affect expression of molecules upstream of mTOR (i.e., protein kinase B [AKT]/tuberous sclerosis [TSC2]). The gene expression of both VDR paralogs (*VDRa* and *VDRb*) was upregulated with an increase in vitamin D levels. *VDRa* expression achieved the highest value in the VD1,573.8 IU/kg group, and *VDRb* in the VD1,980.1 group. Protein expression analysis confirmed that VDR levels exhibited a similar trajectory to vitamin D levels across treatment groups (Fig. 8B). Immunofluorescence revealed increased nuclear translocation of VDR in VD1,167.9 relative to that in the control (Fig. 8C).

## Discussion

### Dietary vitamin D supplementation improves nutrient composition and sensory quality in fish fillets

Muscle is the most important edible tissue in fish and is the main target of nutrient deposition. Indeed, the nutritional value of fish fillets depends on deposition of proteins and lipids in muscle [11]. Here, we addressed the hypothesis that vitamin D can modulate muscle development in fish, thereby influencing flesh quality. To the best of our knowledge, this study is the first to show that dietary vitamin D increases crude protein and lipid contents in fish muscle, improving nutritional value for consumers.

In addition to nutritional value, sensory characteristics represent an important index for evaluating meat quality. For example, a rapid decrease in pH value may lead to undesired denaturation [36]. In the present study, dietary vitamin D supplementation increased pH value and decreased lactic acid content, which was consistent with findings in pigs receiving dietary vitamin D [8]. As reported previously and observed in this study, pH is negatively correlated with lactic acid content [37]. Moreover, levels of cellular lactic acid, the main product of anaerobic metabolism, are negatively correlated with cellular oxygen levels [37]. However, vitamin D maintains mitochondrial activity and integrity, which is a requisite for oxygen-consuming processes such as mitochondrial β-oxidation activities [38]. Indeed, vitamin D deficiency decreases the abundance of hypoxia-inducible factors proteins, which are sensitive to cellular oxygen [39]. Correspondingly, the muscle of fish fed vitamin D in this study exhibited higher pH values and lower lactic acid content compared with that of the un-supplemented control, indicating improved oxygen consumption and attenuated lactic acid production.

Muscle pH influences and myofiber morphology and water-holding capacity, consequently affecting muscle firmness, which can be evaluated via shear force [37]. Our results revealed that the shear force in fish fillets decreased with vitamin D supplementation, indicating a decrease in fillet firmness and improvement in tenderness and quality. Firmness can be further evaluated through collagen content by quantifying the hydroxyproline concentration in fish fillets [11]. In rats, cathepsin B and L catalyze the degradation of collagen [40]. Meanwhile, in fish muscle, collagen glues muscle fibers and fiber bundles together, contributing to the water-holding capacity [41]. Our results suggest that vitamin D supplementation increases collagen levels while decreasing levels of cathepsin B/L, improving water-holding capacity and reducing cooking loss in fillets.

Flavor is an essential element of quality in fish and fish-derived products, directly correlating with consumer acceptance. Amino acids are vital flavor components in meat. Among the amino acids that exhibited significant increases in content under vitamin D supplementation, aspartic acid and glutamic acid contribute to umami, alanine and glycine directly modulate sweetness, and leucine and isoleucine participate in Maillard reactions to form complex aroma compounds [4]. The present study is the first to show that vitamin D can improve the contents of these amino acids in fish muscle, suggesting that dietary vitamin D supplementation can improve the flavor of fish fillets. The antioxidant capacity of fish muscle affects flesh quality [12]. Therefore, we next investigated vitamin D effects on antioxidant capacity in fish muscle.

### Dietary vitamin D supplementation improves antioxidant capacity in fish muscle

Fish flesh is vulnerable to oxidative stress, which caused lower pH value and water holding capacity, resulting in degenerative damage of flesh quality [42]. In the present study, dietary vitamin D significantly reduced the values of ROS, MDA, and PC. The enhanced flesh quality by dietary vitamin D may associated with antioxidant ability in fish muscle. These results were in accordance with a previous report on yellow catfish [18]. ASA and AHR are two key indexes to evaluate the scavenging capacity of free radical (O^2−^ and OH^−^). Our results suggested that vitamin D increased the AHR and ASA activities in the grass carp muscle. Antioxidant system was involved in non-enzymatic compounds (GSH) and antioxidant enzymes. The activities of antioxidant enzymes are closely regulated by corresponding genes [43]. The results showed that dietary vitamin D enhanced GSH content and antioxidant enzymes (GPx isoforms, GST isoforms, GR, GST, CAT, SOD, MnSOD and CuZnSOD), demonstrating that vitamin D could improve the antioxidant ability by altering non-enzymatic antioxidants and antioxidant enzymes in fish muscle. Similar results have been observed in juvenile Chinese mitten crabs fed diets supplemented with vitamin D [19]. Nrf2, which is inhibited by Keap1, plays an important part in regulating antioxidant enzyme-related gene expression [12]. Dietary vitamin D significantly upregulated Nrf2 and decreased Keap1a transcript levels in grass carp muscle, which suggested that dietary vitamin D might improve muscle antioxidant gene expression by regulating the Keap1a-Nrf2 pathway. This phenomenon may be associated with vitamin D metabolites. The structure of vitamin D metabolites (25(OH)_2_D_3_ and 1α,25(OH)_2_D_3_) is similar to that of cell membranes, and thus they protect cell membranes from oxidative stress damage by stabilizing cell structure and reducing membrane fluidity [44]. Besides antioxidant capacity, fish flesh is closely related to muscle growth [13, 14]. Thus, we next deplored the regulation mechanism of vitamin D and muscle growth.

### Dietary vitamin D supplementation improves muscle fiber growth in fish

In fish, flesh firmness is negatively correlated with muscle fiber diameter, which is the primary index of myofiber development [45]. In mice, vitamin D was reported to increase myofiber diameter in skeletal muscle [46]. Similarly, in our experiment, vitamin D supplementation remarkably increased the relative abundance of myofibers with a diameter over 50 μm when compared with control values. The present study therefore demonstrates that dietary vitamin D supplementation during aquaculture induces myofiber growth in fish muscle, thereby improving fillet tenderness.

Mef2 collaborates with MRFs to activate muscle gene expression, playing an essential role at various stages of the differentiation program [23]. In the present study, dietary vitamin D supplementation significantly increased the expression of MyoG, MRF4, Myf5, MyoD, MEF2A, and MEF2C, which may have accounted for the increased average diameter of the muscle fibers. Interestingly, vitamin D had no effect on MEF2B or MEF2D. MEF2B participates in the early processes of muscle development and promotes myogenic cell division [47]. MEF2D regulates the development of muscular cells and the metabolism of microglia, advancing terminal differentiation [48]. These results suggest that vitamin D promotes muscle development during the middle stages of differentiation, but may not be involved in the early and later stages. Overall, our findings indicate that vitamin D improves muscle development by regulating the expression of MRFs and MEF2s, potentially enhancing muscle fiber growth.

### Dietary vitamin D supplementation improves muscle protein deposition

In fish, the hypertrophy stage is strongly associated with fillet protein content, which depends on a balance between protein synthesis and degradation [11]. TOR regulates protein synthesis by suppressing 4E-BP1 and phosphorylating S6K1, which control the translation efficiency of amino acid transport in humans [49]. The present results demonstrate that vitamin D promoted *TOR* and *S6K1* mRNA abundance and reduced that of *4E-BP1* mRNA in fish fillet. Our previously published data showed that vitamin D also plays a key functional role in the absorption and transport of intestinal amino acids [35]. Therefore, the increased in fillet protein content observed here may be associated with improved amino acid absorption in fish intestines and subsequent deposition in muscle. These data suggest that dietary vitamin D regulates muscle protein synthesis via TOR/S6K1/4E-BP1 signaling, thereby enhancing protein content.

A decrease in protein degradation is conducive to protein deposition. The ubiquitin/proteasome-dependent and lysosomal autophagy pathways are components of many cellular regulatory mechanisms involved in protein degradation [50]. Our results revealed that optimal vitamin D supplementation significantly decreased levels of key mRNAs related to protein degradation, along with the protein levels of Beclin 1 and MURF1, indicating that vitamin D may inhibit protein degradation through ubiquitin/proteasome-dependent and lysosomal autophagy pathways.

FoxOs are important factors that directly regulate the transcription of MAFbx and MuRF1 ubiquitin E3 ligase [51]. More specifically, the nuclear localization of FoxO1 and FoxO3 is critical for MAFbx and MurF1 transcription during muscle protein degradation [52]. In this study, vitamin D supplementation was found to decrease the mRNA and protein levels of FoxO1, along with its nuclear localization. These results suggest that vitamin D inhibited protein degradation by FoxO1, rather than by FoxO3, thereby elevating fillet protein content. Our findings showed that vitamin D supplementation can increase fish fillet nutrient composition and improve muscle characteristics through TOR and FoxO1 signaling.

### Dietary vitamin D supplementation improves VDR expression in fish fillet

Akt plays a central role in cell survival by activating or deactivating a series of downstream effector proteins, including TOR [53] and FOXO transcription factors [54]. The AKT-mediated phosphorylation of TSC2 relieves this inhibition to activate mTORC1 [55]. In the present study, dietary vitamin D had no effect on *Akt* or *TSC2* mRNA levels.

mTOR signaling for the enhancement of muscle protein synthesis may be autonomously mediated by the VDR-enhancing anabolic signaling capacity [56]. However, studies have yet to explore the role of vitamin D and VDR paralogs in fish muscle development. Nevertheless, our data showed that vitamin D supplementation increased the expression of *VDRa* and *VDRb*. These data suggest that vitamin D–mediated muscle development in fish somehow involves the two VDR paralogs. Although minimal research has been conducted on the differences between VDR paralogs, The increase in calcium uptake in epithelial calcium channels is regulated by *VDRa* but not *VDRb* in zebrafish [57]. Moreover, *VDRb* (rather than *VDRa*) loss causes reduction and malformation of craniofacial cartilage in zebrafish [58]. Furthermore, a previous study has demonstrated that VDR genes are universally expressed in fish, with a higher transcription abundance of *VDRa* than of *VDRb*. These genes are particularly highly expressed in muscle tissue [59]. This observation supports our conclusion that both genes play important roles in muscle maintenance and development, and elucidation of their specific functions warrants further investigation.

## Conclusion

**Fig. 9:**
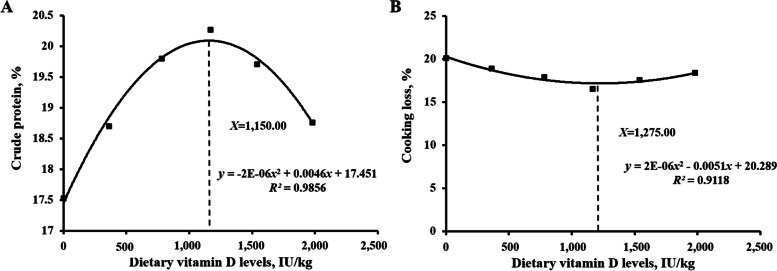
Quadratic regression analysis of crude protein (**A**) and cooking loss (**B**) in the fillets of grass carp (*Ctenopharyngodon idella*) after receiving different levels of dietary vitamin D

To the best of our knowledge, the present study is the first to describe the influence of vitamin D on the nutritional value, sensory quality, antioxidant ability, and myofiber properties of fish fillets through a mechanism associated with the VDR paralogs and the Nrf2, TOR, and FoxO1 signaling pathways. These conclusions were supported by the findings that vitamin D increased nutritional value (crude protein and lipid content), pH, collagen, and hydroxyproline levels in fillets while decreasing shear force, lactate, and cathepsin B/L content. These attributes might be related to the promotion of antioxidant ability and myofiber diameter by vitamin D during muscle fiber growth due to the promotion of differentiation by MRFs, MEF2s, TOR signaling activation, and FoxO1 signaling inhibition. VDR paralogs were also shown to be regulated by dietary vitamin D. Collectively, these data indicate that vitamin D improved flesh quality by promoting muscle development. Based on our comprehensive data regarding crude protein levels and cooking loss, the vitamin D requirements of cultured grass carp (256.89–1,129.67 g) were estimated to be 1,150.00 and 1,275.00 IU/kg, respectively (Fig. 9). This information can be applied to optimize feed parameters and maximize product quality in aquaculture operations. Our study demonstrates the feasibility of nutrient regulation in aquaculture to improve aquatic meat products.

### Supplementary Information

**Additional file 1: Table S1.** Nutritional composition of basal diet. **Table S2.** The analysis method of biomarker and enzymes activity related parameters. **Table S3.** Immunofluorescence staining antibodies. **Table S4.** Real-time PCR primer sequences. **Table S5. **Antibodies for western blot analysis.

## Data Availability

The datasets are included in this article and available from the corresponding author on reasonable request.

## Abbreviations

4E-BP1: eIF4E-binding protein 1
AHR: Antihydroxyl radical
AKT: Protein kinase B
ASA: Anti-superoxide anionl
ATG: Autophagy-related gene
CAT: Catalase
FoxO: Forkhead box O
GPx: Glutathione peroxidase
GR: Glutathione reductase
GSH: Glutathione
GST: Glutathione S-transferase
H&E: Hematoxylin & eosin
Keap1: Kelch-like ECH-associated protein 1
LC3: Light chain 3
MAFbx: Muscle atrophy F-box
MDA: Malondialdehyde
MEF: Myocyte enhancer factor
MRF: Myogenic regulatory factor
MuRF1: Muscle-specific ring finger
Myf5: Myogenic factor 5
MyoD: Myogenic differentiation antigen
MyoG: Myogenin
Nrf2: Nuclear factor erythroid 2-related factor 2
PC: Protein carbonyl
q-PCR: Quantitative polymerase chain reaction
ROS: Reactive oxygen species
S6K1: Ribosomal protein S6 kinase 1
SOD: Superoxide dismutase
T-AOC: Total antioxidant capacity
TOR: Target of rapamycin
TSC2: Tuberous sclerosis 2
UB: Ubiquitin
VDR: Vitamin D receptor
