# Supplementary material for: Implications of vitamin D for flesh quality of grass carp (Ctenopharyngodon idella): antioxidant ability, nutritional value, sensory quality, and myofiber characteristics
Source: J Anim Sci Biotechnol. 2023 Sep 27;14:134. doi: 10.1186/s40104-023-00911-7 (PMC10523690; doi:10.1186/s40104-023-00911-7)
Supplement: Supplementary file 1 — Additional file 1: Table S1. Nutritional composition of basal diet. Table S2. The analysis method of biomarker and enzymes activity related parameters. Table S3. Immunofluorescence staining antibodies. Table S4. Real-time PCR primer sequences. Table S5. Antibodies for western blot analysis. [file 40104_2023_911_MOESM1_ESM.docx]

**Supplementary information**

**Table S1** Nutritional composition of basal diet. (source: Zhang et al., 2022).

| Ingredients (g/kg) Nutrients content (g/kg) ^1^ | | | |
| --- | --- | --- | --- |
| Casein | 135.0 | Crude protein | 300.0 |
| Gelatin | 44.7 | Crude fat | 37.8 |
| Soybean protein concentrate | 220.0 | n-3 PUFAs ^5^ | 10.4 |
| α-starch | 240.0 | n-6 PUFAs | 9.6 |
| Corn starch | 209.1 | n-3: n-6 PUFAs | 10.8 |
| linseed oil | 17.7 | Available phosphorus | 4.0 |
| Soy oil | 14.5 | Vitamin D_3_ (ug/kg) | 3.8 |
| Cellulose | 50.0 |  |  |
| Ca(H_2_PO_4_)_2_ | 14.8 |  |  |
| Vitamin premix ^2^ | 10.0 |  |  |
| Mineral premix ^3^ | 20.0 |  |  |
| Vitamin D_3_ premix ^4^ | 10.0 |  |  |
| Choline chloride (50%) | 10.0 |  |  |
| Ethoxyquin (30%) | 0.5 |  |  |
| DL-Met  In total | 3.7  1000.0 |  |  |

^1^ Crude protein and crude lipid contents represent measured values from air-dried samples. Supplement of available P, n-3 PUFAs, and n-6 PUFAs were calculated according to NRC (2011).

^2^ Per kg of vitamin premix, g/kg: DL -α-tocopherol acetate (50%), 12.58; menadione (22·9%), 0.83; cyanocobalamin (1%), 0.94; D -biotin (2%), 0.75; folic acid (95%), 0.42; thiamine nitrate (98%), 0.09; ascorbyl acetate (95%), 4.31; niacin (99%), 4.04; meso-inositol (98%), 19.39; calcium-D-pantothenate (98%), 3.85; riboflavin (80%), 0.73; pyridoxine hydrochloride (98%), 0.62; retinyl acetate (500,000 IU/g), 2.10. All ingredients were diluted with maize starch to 1 kg.

^3^ Per kg of mineral premix (g/kg): FeSO_4_.H_2_O (30.0% Fe), 12.2500; MgSO_4_.H_2_O (15.0% Mg), 200.0000; ZnSO_4_.H _2_O (34.5% Zn), 8.2460; MnSO_4_.H_2_O (31.8% Mn), 2.6590; CuSO_4_.5H_2_O (25.0% Cu), 0.9560; Na_2_SeO_3_ (44.7% Se), 0.0168; KI (76.9% I), 0.0650 g. All ingredients were diluted with maize starch to 1 kg.

^4^ Vitamin D_3_ premix: premix was added to obtain graded levels of vitamin D_3_ and the amount of maize starch was reduced to compensate.

^5^ PUFAs = polyunsaturated fatty acids

Table S2 The analysis method of biomarker and enzymes activity related parameters

| Indices | Commercial kit | Catalog number | Detection range |
| --- | --- | --- | --- |
| Reactive oxygen species (ROS) | Nanjing Jiancheng commercial assay kits (Nanjing, China) | E004 | No detection range |
| Malondialdehyde (MDA) | Nanjing Jiancheng commercial assay kits (Nanjing, China) | A003–1 | 0-113.0 nmol/mL |
| Protein carbonyl (PC) | Nanjing Jiancheng commercial assay kits (Nanjing, China) | A087 | 1-1100 nmol/L |
| Total antioxidant capacity (T-AOC) | Nanjing Jiancheng commercial assay kits (Nanjing, China) | A015 | 0.2-55.2 U/ml |
| Anti-superoxide anion (ASA) | Nanjing Jiancheng commercial assay kits (Nanjing, China) | A052 | 0.5-250 U/L |
| Anti-hydroxy radical (AHR) | Nanjing Jiancheng commercial assay kits (Nanjing, China) | A018 | 10.1-736.7 U/mL |
| Superoxide dismutase (SOD) | Nanjing Jiancheng commercial assay kits (Nanjing, China) | A001–3 | 0.5-15 U/mL |
| Catalase (CAT) | Nanjing Jiancheng commercial assay kits (Nanjing, China) | A007 | 0.2-24.8 U/mL |
| Glutathione peroxidase (GPx) | Nanjing Jiancheng commercial assay kits (Nanjing, China) | A005 | 20U-330 U |
| Glutathione-S-transferase (GST) | Nanjing Jiancheng commercial assay kits (Nanjing, China) | A004 | 6.0-22.0 U/mL |
| Glutathione reductase (GR) | Nanjing Jiancheng commercial assay kits (Nanjing, China) | A062 | 1.6-321.5 U/L |
| Glutathione (GSH) | Nanjing Jiancheng commercial assay kits (Nanjing, China) | A006–2 | 0.1-100 umol/L |
| Protein concentrations | Nanjing Jiancheng commercial assay kits (Nanjing, China) | A045–2-2 | 0.05-1.3 mg/ml |

**Reference：**

Zhang Y, Li CN, Jiang WD, Wu P, Liu Y, Kuang SY, et al. An emerging role of vitamin D_3_ in amino acid absorption in different intestinal segments of on-growing grass carp (*Ctenopharyngodon idella*). Anim Nutr. 2022;10:305-18. https://doi.org/10.1016/j.aninu.2022.05.004

**Table S3** Immunofluorescence staining antibodies.

| Indices^1^ | Host | Source | Catalog No. | Dilution |
| --- | --- | --- | --- | --- |
| FoxO1  VDR  Alexa Fluor 488  Alexa Fluor 555 | Rabbit  Rabbit  Rabbit  Rabbit | Affinity (Golden, Colorado, USA)  Abclonal (Wuhan, Hubei, CN)  Beyotime (Nanjing, Jiangsu, CN)  Beyotime (Nanjing, Jiangsu, CN) | AF6416  A2194  A0423  P0179 | 1:300  1:300  1:1000  1:1000 |

^1^ FoxO1, forkhead box O1; VDR, vitamin D receptor.

**Table S4** Real-time PCR primer sequences.

| Target Gene^1^ | Primer sequence Forward (5′→ 3′) | Primer sequence Reverse (5′ → 3′) | **Amplification efficiency (%)** | **product size (bp)** | Accession number |
| --- | --- | --- | --- | --- | --- |
| *CuZnSOD* | CGCACTTCAACCCTTACA | ACTTTCCTCATTGCCTCC | 100.3 | 218 | GU901214 |
| *MnSOD* | ACGACCCAAGTCTCCCTA | ACCCTGTGGTTCTCCTCC | 99.2 | 111 | GU218534 |
| *CAT* | GAAGTTCTACACCGATGAGG | CCAGAAATCCCAAACCAT | 100 | 157 | FJ560431 |
| *GPx1a* | GGGCTGGTTATTCTGGGC | AGGCGATGTCATTCCTGTTC | 100 | 201 | EU828796 |
| *GPx1b* | TTTTGTCCTTGAAGTATGTCCGTC | GGGTCGTTCATAAAGGGCATT | 100 | 163 | KT757315 |
| *GPx4a* | TACGCTGAGAGAGGTTTACACAT | CTTTTCCATTGGGTTGTTCC | 99.9 | 196 | KU255598 |
| *GPx4b* | CTGGAGAAATACAGGGGTTACG | CTCCTGCTTTCCGAACTGGT | 100.1 | 208 | KU255599 |
| *GSTR* | TCTCAAGGAACCCGTCTG | CCAAGTATCCGTCCCACA | 99.6 | 208 | EU107283 |
| *GSTp1* | ACAGTTGCCCAAGTTCCAG | CCTCACAGTCGTTTTTTCCA | 100 | 107 | KM112099 |
| *GSTp2* | TGCCTTGAAGATTATGCTGG | GCTGGCTTTTATTTCACCCT | 100 | 94 | KP125490 |
| *GSTo1* | GGTGCTCAATGCCAAGGGAA | CTCAAACGGGTCGGATGGAA | 100.2 | 208 | KT757314 |
| *GSTo2* | CTGCTCCCATCAGACCCATTT | TCTCCCCTTTTCTTGCCCATA | 99.9 | 110 | KU245630 |
| *GR* | GTGTCCAACTTCTCCTGTG | ACTCTGGGGTCCAAAACG | 99.5 | 221 | JX854448 |
| *Nrf2* | CTGGACGAGGAGACTGGA | ATCTGTGGTAGGTGGAAC | 100.6 | 234 | KF733814 |
| *Keap1a* | TTCCACGCCCTCCTCAA | TGTACCCTCCCGCTATG | 100.2 | 205 | KF811013 |
| *Keap1b* | TCTGCTGTATGCGGTGGGC | CTCCTCCATTCATCTTTCTCG | 99 | 87 | KJ729125 |
| *Myf5* | GGAGAGCCGCCACTATGA | GCAGTCAACCATGCTTTCAG | 93.7 | 257 | MG574982 |
| *MyoG* | AGAGGAGGTTGAAGAATTTC | GTTCCTGCTGGTTGAGAGA | 95.5 | 178 | JQ793897 |
| *MRF4* | GAAAATCTGCTCCAACTACC | CGCTGCGTAAAATCTCCA | 105.3 | 152 | JQ793896 |
| *MEF2A* | ACATTCACACATCCACAAGA | GCACTGCTCAGGGAGTATTC | 99.7 | 102 | / |
| *MEF2B* | TCGCTCTCATCATCTTCAAC | AGCCTCCAGTATGTCAGTGT | 100.2 | 134 | XM051909340 |
| *MEF2C* | CGGCTTCTCTTCATCTGGGT | GCTCTGACATAACTGTGTGCTG | 99.4 | 104 | / |
| *MEF2D* | AGCACTCAGATGGTGAACAG | ATGGAGAATGGAGTCAGGAG | 99.7 | 173 | XM51864640 |
| *TOR* | TCCCACTTTCCACCAACT | ACACCTCCACCTTCTCCA | 100 | 177 | JX854449 |
| *S6K1* | TGGAGGAGGTAATGGACG | ACATAAAGCAGCCTGACG | 100.4 | 111 | EF373673 |
| *4E-BP1* | GCTGGCTGAGTTTGTGGTTG | CGAGTCGTGCTAAAAAGGGTC | 100.7 | 164 | KT757305 |
| *MuRF1* | TGTCTATGGACTACAGAGGAA | GGATTTCAAAGGAGGTTCAAG | 98.6 | 103 | / |
| *MAFbx* | CGGACGAGATCTGGTTAGCC | GCTTGCGGATCTGTCTGTCT | 91.2 | 119 | / |
| *ATG5* | GGAGGAGATGTGGTTTGAGTATG | GCCCAGAACTGGTCGAATTTA | 95.5 | 161 | MK635464 |
| *ATG7* | TTTGAAGACTGTCTGAGCGG | GCCATTGTCAACTCGGAAAAG | 105.4 | 149 | MG797681 |
| *Beclin-1* | AGCTCGACACATCCTTCAAC | CTGCGACTCAAGTTCTCCATAG | 101.7 | 157 | MN311523 |
| *LC3-Ⅰ* | GCCCAAACAAGGCAACATAC | TGACAAGACGATACGCAACTC | 91.3 | 104 | / |
| *LC3-Ⅱ* | AGCAGCGGGTGGAGGATGTA | CCTCAGAAATGGCGGTGGAC | 94.4 | 153 | KU886023 |
| *UB* | GCCAAGCGACACCATTGAG | GGATGTTGTAGTCGGACAG | 105.6 | 131 | / |
| *FOXO1a* | GCATCTCATAGCCATGCCCT | CACCTCCAAGATGACCGGAG | 107.8 | 190 | KP325483 |
| *FOXO1b* | CTCAACCTCATCTCGCCCAA | TCGGTATGGCGATTGGACTG | 102.5 | 196 | KP325484 |
| *FOXO3a* | GCTGCGTAGTGATCCCATGATGTC | GGAGACTGTTGGAGATGCTGCTTC | 92.1 | 144 | / |
| *AKT* | CCTGGTGATGAAGGAGCTGA | CTGTCAGAGAGCCTCCAGCA | 97.7 | 113 | KY763985 |
| *TSC2* | CAATGAAGCCAACAAACCCC | GTGAGGAACTGTGCGTAGCG | 98.6 | 192 | / |
| *VDRa* | CAGAGAGTCGTACCGTTTCG | ACAAGTCTTTGCCTTTCAGC | 100 | 248 | MW789222 |
| *VDRb* | ATTGACACGCTGGTGGATGC | CTGGAGAGTGGCTGAAGGAGTC | 99.9 | 169 | MW789223 |
| β-actin | GGCTGTGCTGTCCCTGTA | GGGCATAACCCTCGTAGAT | 100 | 101 | M25013 |

^1^ SOD, superoxide dismutase; CAT, catalase; GPx, glutathione peroxidase; GR, glutathione reductase; GST, glutathione S-transferase; Nrf2, NF-E2-related factor 2; MyoD, myogenic differentiation antigen; Myf5, myogenic factor 5; MyoG, myogenin; MRF4, myogenic regulatory factor 4; MEF: myocyte enhancer factor; TOR, target of rapamycin; S6K1, ribosomal protein S6 kinase; 4E-BP1, eIF4E-binding protein 1; MuRF1, muscle-specific ring finger; MAFbx, muscle atrophy F-box; ATG, autophagy; LC3, light chain; UB, ubiquitin; FoxO, forkhead box O; AKT, protein kinase B; TSC2, tuberous sclerosis 2.

**Table S5** Antibodies for western blot analysis.

| Indices | Host | Source | Catalog No. | Dilution |
| --- | --- | --- | --- | --- |
| MyoG | Rabbit | Affinity (Golden, Colorado, USA) | DF8273 | 1:1000 |
| MRF4 | Rabbit | Abcam (Cambridge, MA, USA) | Ab32550 | 1:1200 |
| Myf5 | Rabbit | Abclonal (Wuhan, Hubei,CN) | A16227 | 1:1200 |
| MyoD | Rabbit | Affinity (Golden, Colorado, USA) | AF7733 | 1:1500 |
| MEF2A | Rabbit | Abcam (Cambridge, MA, USA) | Ab76063 | 1:1300 |
| MEF2C  T-TOR  p-TORSer488  T-S6K1  p-S6K1Ser389  T-4E-BP1  p-4E-BP1Thr37/46  β-actin  Beclin 1  MuRF1  FoxO1  UB  VDR  Secondary antibody | Rabbit  Rabbit  Rabbit  Rabbit  Rabbit  Rabbit  Rabbit  Rabbit  Rabbit  Rabbit  Rabbit  Rabbit  Rabbit  Rabbit | Abcam (Cambridge, MA, USA)  Affinity (Golden, Colorado, USA)  Affinity (Golden, Colorado, USA)  Affinity (Golden, Colorado, USA)  Affinity (Golden, Colorado, USA)  Affinity (Golden, Colorado, USA)  Affinity (Golden, Colorado, USA)  Affinity (Golden, Colorado, USA)  Abcam (Cambridge, MA, USA)  Abcam (Cambridge, MA, USA)  Abclonal (Wuhan, Hubei,CN)  Abclonal (Wuhan, Hubei,CN)  Abclonal (Wuhan, Hubei,CN)  Affinity (Golden, Colorado, USA) | Ab227085  AF6308  AF3308  AF6226  AF3228  AF6432  AF3830  AF7018  Ab62557  ab183094  A2934  A18185  A2194  S0001 | 1:1300  1:1000  1:1000  1:1500  1:1300  1:1500  1:1200  1:3000  1:1500  1:1500  1:1000  1:1000  1:800  1:8000 |
